# Advancing dermoscopy through a synthetic hair benchmark dataset and deep learning-based hair removal

**DOI:** 10.1117/1.JBO.29.11.116003

**Published:** 2024-11-19

**Authors:** Lennart Jütte, Harshkumar Patel, Bernhard Roth

**Affiliations:** a Leibniz University Hannover, Hannover Centre for Optical Technologies, Hannover, Germany; b Leibniz University Hannover, Cluster of Excellence PhoenixD, Hannover, Germany

**Keywords:** synthetic hair dataset, deep learning, dermoscopy, skin cancer, melanoma

## Abstract

**Significance:**

Early detection of melanoma is crucial for improving patient outcomes, and dermoscopy is a critical tool for this purpose. However, hair presence in dermoscopic images can obscure important features, complicating the diagnostic process. Enhancing image clarity by removing hair without compromising lesion integrity can significantly aid dermatologists in accurate melanoma detection.

**Aim:**

We aim to develop a novel synthetic hair dermoscopic image dataset and a deep learning model specifically designed for hair removal in melanoma dermoscopy images.

**Approach:**

To address the challenge of hair in dermoscopic images, we created a comprehensive synthetic hair dataset that simulates various hair types and dimensions over melanoma lesions. We then designed a convolutional neural network (CNN)-based model that focuses on effective hair removal while preserving the integrity of the melanoma lesions.

**Results:**

The CNN-based model demonstrated significant improvements in the clarity and diagnostic utility of dermoscopic images. The enhanced images provided by our model offer a valuable tool for the dermatological community, aiding in more accurate and efficient melanoma detection.

**Conclusions:**

The introduction of our synthetic hair dermoscopic image dataset and CNN-based model represents a significant advancement in medical image analysis for melanoma detection. By effectively removing hair from dermoscopic images while preserving lesion details, our approach enhances diagnostic accuracy and supports early melanoma detection efforts.

## 1 Introduction

The early detection and diagnosis of melanoma, a skin cancer originating from melanocytes, remains a pivotal challenge in dermatology.^1^ Melanoma is treatable in its early stages but metastasizes to other parts of the body in later stages, making it one of the deadliest forms of skin cancer.^2^ The incidence of melanoma has been rising steadily over the past decades, becoming a significant public health concern globally.^3^ The gold standard for diagnosing melanoma is histopathology which is performed on an excised sample of the lesion. Dermoscopy, a non-invasive skin examination technique, is the established imaging tool in the early detection of melanoma, providing dermatologists with enhanced visualization of subsurface skin structures that are not visible to the naked eye. This technique significantly increases the accuracy of melanoma diagnoses by enabling the detailed observation of characteristic features of melanoma lesions.^4^ However, the presence of hair in dermoscopic images presents a considerable challenge, often obscuring critical details of the skin lesions and potentially leading to misdiagnosis or decreasing the confidence of dermatologists in their diagnosis.^5^

The intersection of medical imaging and computational technology, particularly through the application of deep learning algorithms, offers an opportunity to address the challenges posed by hair in dermoscopic images. Deep learning has shown exceptional progress in various tasks of image analysis, including recognition, classification, and segmentation. In dermatology, deep learning models have been developed for the automated analysis of dermoscopic images, aiming to enhance the accuracy and efficiency of melanoma detection.^6^

In the following, we summarize the state of the art in hair removal techniques for dermoscopic images, highlighting significant contributions and their impact on the field. Several methods have been proposed and developed over the years to address this challenge. Lee et al.^7^ introduced Dullrazor, a pioneering software approach that employs morphological operations and median filtering to remove hair artifacts from images. This method has been widely used due to its effectiveness at the time in preserving important image features. Xie et al.^8^ proposed a partial differential equation (PDE)-based method for repairing hair-occluded information in dermoscopy images, focusing on mathematical modeling to reconstruct occluded areas and improve image quality. Abbas et al.^9^ conducted a comparative study of different hair removal methods, evaluating their performance and providing insights into the strengths and weaknesses of each technique. This study highlighted the importance of selecting appropriate methods for different types of dermoscopy images. Huang et al.^10^ developed a robust hair segmentation and removal approach using edge detection and region-growing techniques, which proved effective in clinical settings. Bibiloni et al.^11^ presented a novel method utilizing soft color morphology for hair removal in dermoscopic images. Deep learning techniques have become state-of-the-art for various computer vision tasks, including image restoration. These methods learn network parameters to reconstruct images from training data comprising clean and corrupted image pairs. Xie et al.^12^ combined sparse coding with pre-trained deep networks for image denoising and blind inpainting, achieving notable results. Vincent et al.^13^ introduced a stack of denoising autoencoders for image denoising, applied recursively to intermediate representations to initialize deep networks. Overall, the development of these methods underscores the evolving landscape of hair removal techniques in dermoscopic image processing. The diversity in skin tones and hair colors can present different challenges in the hair removal process, and it is important to ensure that the model performs consistently across these variations. Dark hair on light skin typically has high contrast, making it easier for any model and classical image processing techniques to detect and remove. However, lighter hair (blonde, gray) or hair on light and darker skin presents a challenge due to lower contrast, which can complicate segmentation and removal.

Our work focuses on two main innovations: the creation of a diverse synthetic hair dermoscopic image benchmark dataset and the development of a deep learning model specifically designed for the removal of hair from dermoscopic images of melanoma lesions. The synthetic hair dataset aims to simulate a wide range of hair types, colors, and densities overlaying melanoma lesions, providing a novel resource for training and evaluating hair removal algorithms. The creation of a synthetic hair image benchmark dataset addresses the need for standardized and diverse resources to facilitate the advancement of hair removal techniques. Furthermore, the development of a deep learning model for hair removal directly confronts the challenge of hair occlusion, aiming to improve the clarity and diagnostic utility of dermoscopic images, thereby enhancing the accuracy of melanoma diagnoses. The dataset creation involved a combination of algorithmic frameworks for hair mask generation and hair simulation, ensuring a diverse and realistic representation of hair artifacts in dermoscopic images. The deep learning model, leveraging convolutional neural networks (CNNs), was specifically tailored for hair removal in dermoscopic images. The model’s architecture was designed to balance the need for effective hair removal with the preservation of crucial lesion details, critical for accurate melanoma diagnosis.

The use of a synthetic dataset is driven by the constraint that there are currently no available dermoscopic datasets of sufficient size that include images both with hair and with the hair physically removed (e.g., by shaving). Because our approach requires ground-truth images without hair for accurate training and evaluation, a synthetic dataset is the most viable option. The synthetic dataset allows for the generation of paired images (with and without hair) under controlled conditions, which is essential for training the deep learning model effectively. Furthermore, this approach allows for the simulation of a wide range of hair conditions and patterns, ensuring that the model can generalize well to diverse clinical scenarios.

The synthetic dataset allows us to precisely control the characteristics of the hair (such as length, thickness, and density), which would be difficult to standardize across real-world datasets. This control enables a more systematic evaluation of the model’s ability to remove hair artifacts under varying conditions. Synthetic data enables us to simulate challenging clinical conditions, such as unusual hair patterns or dense hair coverage, which may be less frequently encountered in real-world datasets. This ensures that the model is robust across a wide range of scenarios, including those that are less common but clinically relevant.

Thus, our paper addresses a significant challenge in the analysis of dermoscopic images and aims to enhance the precision of melanoma diagnosis through the creation of a synthetic hair image benchmark dataset and the development of a deep learning model for hair removal. The tool is intended for use in specific cases in which hair poses a challenge to melanoma detection. As only publically available datasets have been utilized, the patient privacy is maintained in this approach.

## 2 Methods

### 2.1 Creation of Synthetic Hair Dataset

In the first step, we developed the synthetic hair image benchmark dataset, designed to simulate a broad spectrum of hair types, colors, and densities overlaying melanoma lesions. The dataset’s creation involved a structured algorithmic framework that combined image processing techniques in MATLAB with advanced simulation methods in Python.

We utilized MATLAB’s extensive image processing capabilities to generate hair masks.^14^ An algorithm was developed to create detailed hair masks that accurately represent various hair characteristics, including thickness, length, and number of hairs. A custom script generates these masks by setting parameters such as the number of hair strands and their dimensions, using bézier curves^15^ to simulate natural hair curvature. The script introduces variability and realism through specific algorithms, including anti-aliasing techniques^16^ to smooth hair edges and Gaussian blur^17^ to enhance the hair mask quality, mimicking real dermoscopic images.

**Fig. 1:**
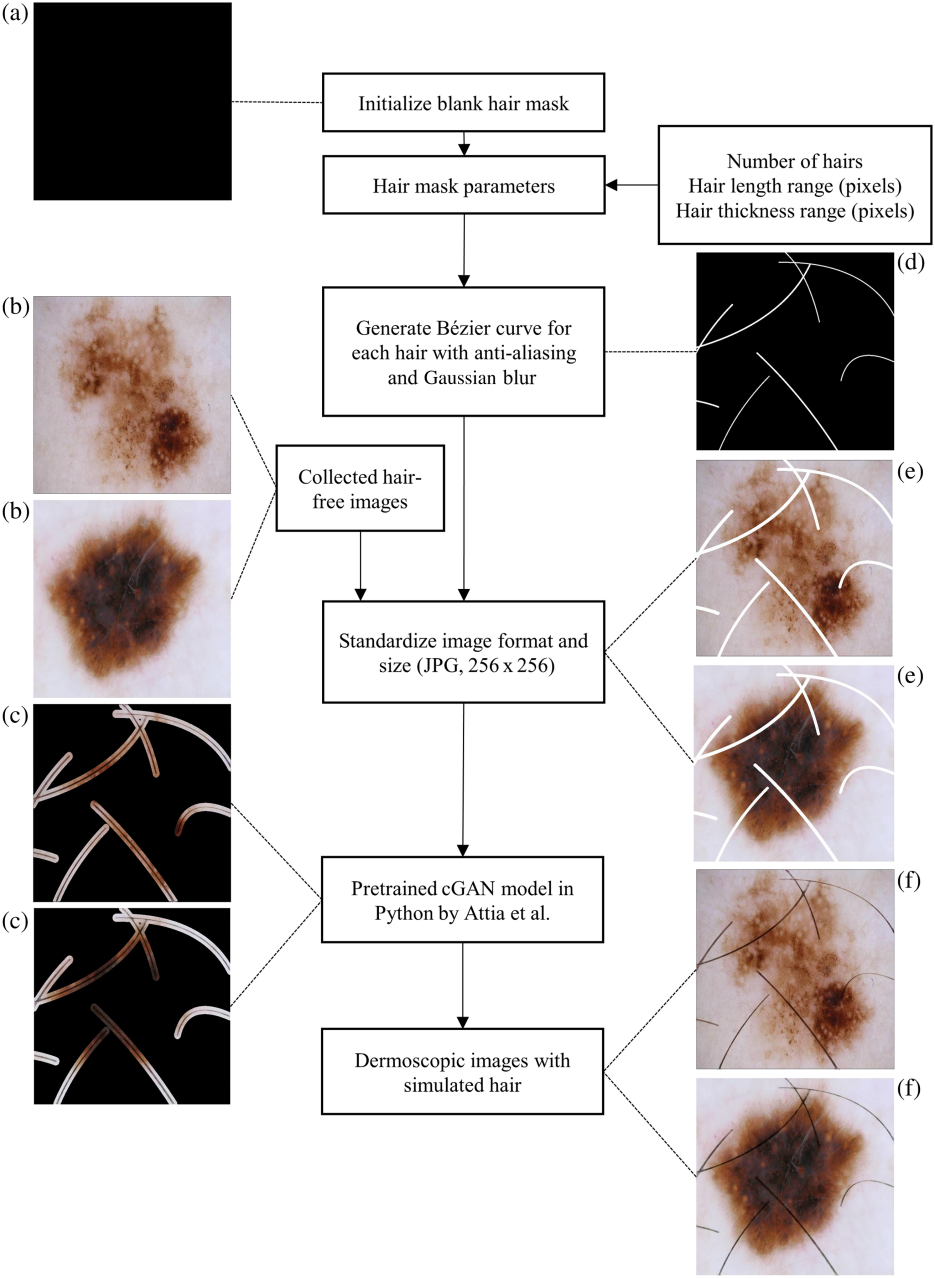
Process flowchart for creating synthetic hair images based on Bézier curve hair masks and a pre-trained cGAN. (a) blank hair mask, (b) ground-truth images, (c) focus areas of the model for the hair generation, (d) hair mask, (e) hair mask on the ground-truth images, and (f) generated synthetic hair image.

These masks served as templates for the subsequent simulation of hair on dermoscopic images of skin lesions. The flowchart in Fig. 1 illustrates the entire process of generating synthetic hair, from the initial creation of hair masks in MATLAB to their application in simulating realistic hair patterns on images of skin lesions.

Following the creation of hair masks, Python’s machine learning libraries, specifically PyTorch, torchvision, and OpenCV, were employed to simulate hair on dermoscopic images based on the previously generated masks. We utilized a pre-trained model by Attia et al.^18^ Our process unfolds in three primary steps: data preparation, hair synthesis, and hair merging. In data preparation, 1.064 hair-free dermoscopic images are manually selected, with hair masks applied to delineate areas for synthetic hair application, ensuring the integrity of underlying skin details. During hair synthesis, the MATLAB-generated masks guide the Generative Adversarial Network (GAN) in accurately placing synthetic hair on marked regions of the images. The hair merging stage then seamlessly integrates synthetic hair into the original images, preserving the diagnostic features of the skin lesions.^18^

The process for creating a synthetic hair dataset in dermoscopic images follows several methodical steps within MATLAB. The process begins with creating an empty mask to act as the canvas for drawing the synthetic hair strands. This mask is a matrix initialized to zeros, which will be populated with the pixel values representing the hairs. Afterward, parameters such as the number of hairs, their length, and thickness are predefined. These parameters control the properties of the generated hairs, ensuring variability to mimic real hair in dermoscopic images. Anti-aliasing is applied to the hair strands as they are drawn to prevent jagged edges, which could otherwise occur due to pixelation in the digital environment. This ensures that the strands look smooth and realistic. A Bézier curve is used to create each hair strand. The Bézier curve is generated based on four control points, and a parameter that moves the point along the curve, giving each strand a natural curve shape. Gaussian blur is applied to the hair strands to simulate the softness of natural hairs, ensuring the edges are not overly sharp and mimic real-world optical effects, especially in out-of-focus areas. After applying Gaussian blur and completing the drawing of all hair strands, the final hair mask is saved as a digital image. This mask can be overlaid onto dermoscopic images for analysis or further processing.

This application of pre-trained models aims to mimic the appearance of real hair, including its texture, color variation, and natural randomness, to achieve high realism in the synthetic images, ensuring they closely resemble actual dermoscopic images with hair artifacts.

### 2.2 Development of Deep Learning Model for Hair Removal

The developed CNN architecture is tailored specifically for the task of hair removal in dermoscopic images. The model was structured in MATLAB to balance the two objectives of effectively removing hair artifacts and preserving essential details of the melanoma lesions.

#### 2.2.1 Model architecture and training

We have adopted a U-Net-like architecture, known for its effectiveness in image-to-image translation tasks, particularly suited for detailed feature manipulation while preserving the overall structure of the image.^19^ This section outlines the layered structure of our model, emphasizing the integration of skip connections for enhanced performance. The encoder path in our CNN plays a crucial role in analyzing and downsampling the input images to capture essential features at varying scales. It begins with convolutional layers with 3 × 3 filters, which identify low-level features such as edges and textures. The decoder path inverses the encoder’s process by methodically enhancing the spatial dimensions of the feature maps to reinstate their original size through transposed convolutional layers.^20^ Additional convolutional layers refine the upscaled features, with skip connections from the encoder improving the decoder’s outputs by including detailed features with broader semantic content. This blending is important for preserving lesion details in the output images.

**Fig. 2:**
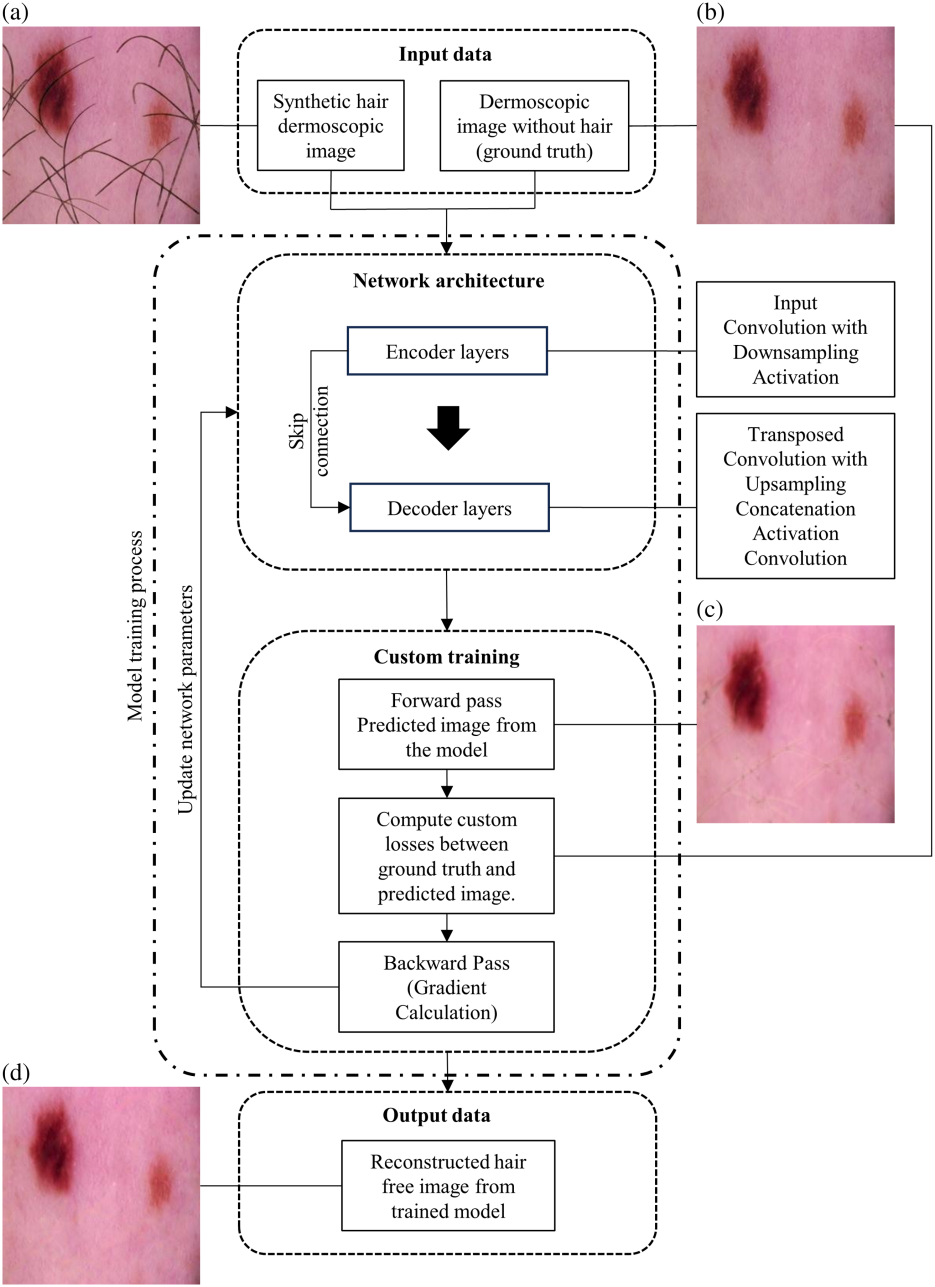
Process flowchart for digital hair removal deep learning model in dermoscopic images and the model training process. (a) Generated synthetic hair dermoscopic image, (b) ground-truth image, (c) model prediction for loss evaluation, and (d) final model output dermoscopic image after digital hair removal.

The skip connections are preserving spatial information by reintegrating bypassed information into deeper layers of the network, preventing the loss of fine-grained details.^21^ The output layer, a convolutional layer with 1×1 filters, translates the feature maps into the final image, reflecting the targeted changes while maintaining the input dimensions. The architecture processes input images of size 256×256  pixels in the RGB color space, effectively handling high-resolution images. The process is illustrated in Fig. 2.

#### 2.2.2 Model training and optimization

The training of the model involved the dataset comprising synthetic hair dermoscopic images alongside the corresponding actual dermoscopic images without hair artifacts. A custom training loop was implemented, providing precise control over the model’s training iterations. The training was conducted using a dataset consisting of 852 paired images. This manual approach allowed for the integration of custom operations during the forward and backward passes. The forward pass computes the network’s outputs for given inputs, whereas the backward pass involves backpropagation, in which gradients are calculated for each parameter, informing how they should be updated to minimize loss.

The model employs the Adam optimizer with a learning rate of 0.0001, optimizing the combined loss to enhance the coherence of the hairless output images. A combination of loss functions was employed to optimize the model’s performance. This multi-faceted approach to loss calculation ensured that the model not only effectively removed hair but also maintained the integrity of lesion details critical for accurate diagnosis. To optimize performance, a composite loss function was used, including

$$L_{\text{total}}=\lambda_{1}L_{\text{hair}}+\lambda_{2}L_{\text{non}-\mathrm{hair}}+\lambda_{3}L_{\text{normalized}}+\lambda_{4}L_{\mathrm{SSIM}}+\lambda_{5}L_{\text{total variation}}$$ (1)

where $\lambda_{1}$, $\lambda_{2}$, $\lambda_{3}$, $\lambda_{4}$, and $\lambda_{5}$ are weights assigned to each loss component, respectively. The following losses have been implemented:

• Hair pixel loss ($L_{\text{hair}}$): Targets hair pixels specifically, ensuring they are effectively replaced by inpainting. This uses mean squared error (MSE)^22^ to quantify the difference between the predicted inpainted areas and the actual image data in the hair regions.
• Non-hair pixel loss ($L_{\text{non}-\mathrm{hair}}$) focuses on non-hair areas, ensuring that inpainting maintains natural appearances and textures.
• Normalized hair pixel loss ($L_{\text{normalized}}$): This loss function focuses on minimizing errors in hair regions relative to the entire image, ensuring a balance between the fidelity of hair texture reconstruction and overall image quality.
• Structural similarity index (SSIM) loss ($L_{\mathrm{SSIM}}$) ensures that the inpainted regions maintain structural and textural consistency with the rest of the image.^23^
• Total variation loss ($L_{\text{total variation}}$) encourages smooth transitions in the inpainted areas, reducing noise and preserving edges.^24^

This robust training approach aims to ensure that the network efficiently learns to perform high-quality hair removal while preserving critical details necessary for accurate dermatological diagnosis.

### 2.3 Evaluation of Model Performance

The model’s performance was evaluated against the synthetic hair image benchmark dataset using a comprehensive set of metrics, including structural similarity index measure (SSIM),^23^ MSE,^22^ mean absolute error (MAE),^22^ and multi-scale structural similarity index measure (MSSSIM).^25^ These metrics, widely recognized for their effectiveness in assessing image quality, provided a detailed assessment of the model’s ability to remove hair without compromising the diagnostic features of melanoma lesions. Values closer to 1 for SSIM and MSSSIM indicate superior performance, highlighting the model’s capacity to maintain structural and textural accuracy in hair-removed images. The MSE and MAE should be as small as possible.

## 3 Results

This section shows the outcomes of the implementation of the synthetic hair dataset and the deep learning model for hair removal, focusing on their performance, accuracy, and utility in enhancing the clarity of melanoma dermoscopic images.

### 3.1 Synthetic Hair Dataset Development

The creation of the synthetic hair dataset involved a combination of algorithmic frameworks and simulation techniques, using Bezier curves to generate detailed hair masks. These masks, designed to capture the characteristics of real hair more accurately than initial attempts or publicly available real hair masks, were created in MATLAB with specific parameters: hair length ranging from 100 to 900 pixels, number of hairs set from 1 to 40, and thickness varying from 1 to 4 pixels. This precise parameterization facilitated the production of 1064 diverse hair masks, leading to a dataset of 1064 synthetic hair dermoscopic images that closely resemble real hair in terms of texture, density, and natural variability. Eighty percent of the images were used for training the model, and 20% were reserved exclusively for testing. These subsets were created in such a way that no image in the testing set was used during the training process.

**Fig. 3:**
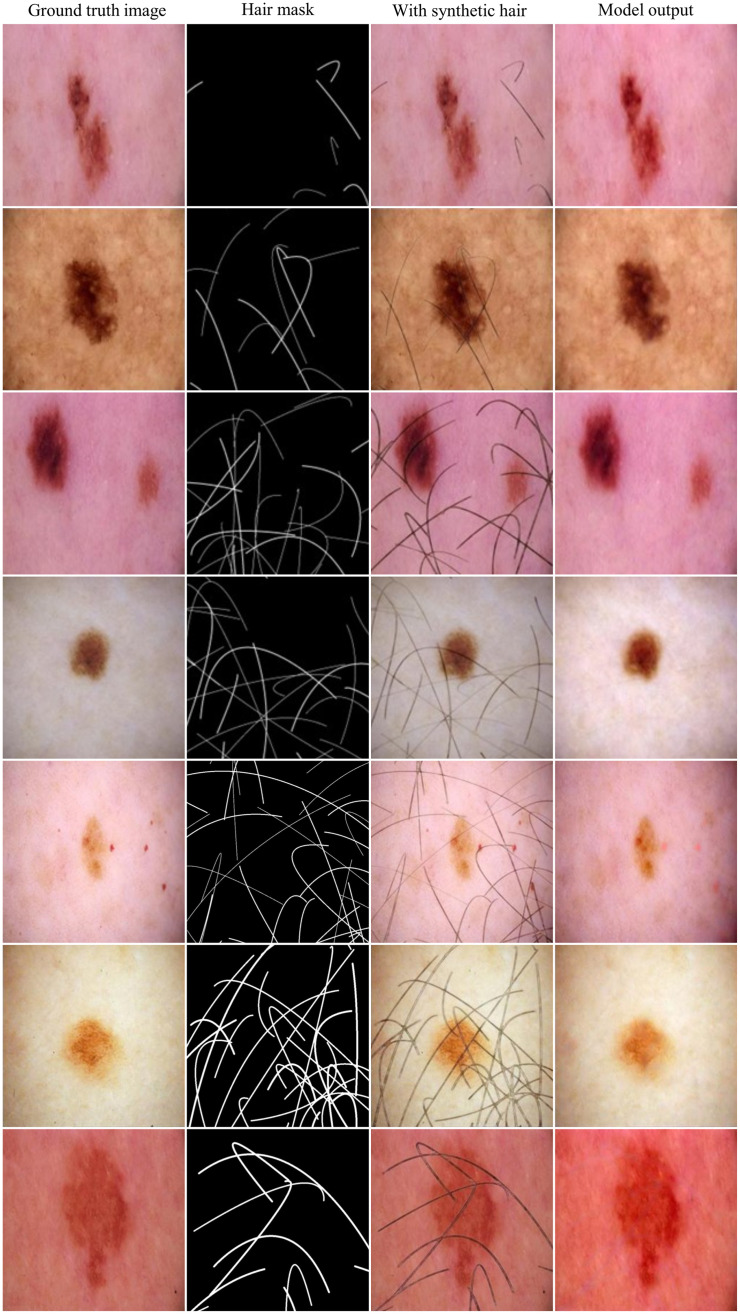
Comparison of the synthetic hair-generated masks and images using Bézier curves and a pretrained cGAN model, and the model output. The ground truth images stem from the ISIC dataset.

This dataset, overlaying simulated hair on a diverse collection of dermoscopic images of melanoma lesions, represents a broad array of hair types, colors, and densities, set against varied skin tones and lesion types. Such diversity is important for the robust training and evaluation of hair removal algorithms, ensuring their effectiveness across different clinical situations. The high degree of diversity and realism in the synthetic hair dataset enhances its utility as a resource for developers of dermatological diagnostic tools. Figure 3 presents a comparative analysis of image pairs showing the synthetic hair generation process and the model output.

The results demonstrate that the model robustly removes hair while preserving the clarity and integrity of the underlying skin lesions. In summary, the development of this dataset resulted in the integration of custom-generated Bezier hair masks with a pre-trained model for synthetic hair generation. This approach has significantly simplified the creation process, allowing for scalability and enhanced diversity in the dataset.

The dataset includes a broad range of skin tones, covering Fitzpatrick Types I (very light) to VI (very dark), to ensure inclusivity across different skin types. The specific distribution is as follows: types I–II, 6.39%; types III–IV, 89.76%; and types V–VI, 3.85%. Although the majority of the images represent types III–IV, all skin types are represented, ensuring that the model is exposed to a diverse spectrum of skin tones. This diversity is critical for training the model to perform well across various clinical scenarios, especially because types III and IV are more commonly encountered in dermoscopic datasets, whereas the inclusion of very light and very dark skin types ensures the model can generalize well to less common, but clinically significant cases.

The variety of hairs is simulated to reflect real-world conditions. The color of the hair is automatically taken from the base image color. The model ensures contrast between the hair and the skin tone. The hair density in our dataset ranges from 1 to 40 strands per image, with hair lengths spanning from 100 to 900 pixels, and thicknesses varying between 1 and 4 pixels. Each of these metrics follows a uniform distribution, ensuring variability and randomness in the hair patterns, which cover all real-world conditions. The hair patterns are designed to replicate the natural randomness and variability found in real dermoscopic images. Low-density images (1 to 5 hairs) make up 12.5% of the dataset, medium-density images (6 to 20 hairs) account for 37.5%, and high-density images (21 to 40 hairs) represent 50%. The lower percentage of images with a small number of hairs (1 to 5 hairs) in the dataset is intentional. In clinical practice, dermatologists typically do not face significant challenges when dealing with images containing only a few hairs, as these minimally obstruct the view of the lesion. The difficulties arise with images that contain a higher density of hair (6 to 20 and especially 21 to 40 hairs), in which the hair coverage can obscure important diagnostic features of the lesion, such as borders and pigmentation patterns. As a result, the dataset is designed to reflect this practical reality, with a greater emphasis on images containing more hair. This ensures that our model is better suited for situations where hair obstruction presents a real challenge to accurate melanoma detection. The model’s performance is, therefore, optimized for scenarios where hair density is high, addressing the needs of dermatologists in the most critical cases. By including this wide range of skin tones, hair colors, densities, and lengths, we aimed to create a comprehensive dataset that reflects the variability seen in real clinical settings. This diversity allows our model to generalize better across different populations and conditions.

### 3.2 Deep Learning Model for Hair Removal

The deep learning model developed for hair removal was extensively tested against the synthetic hair dataset. The model’s performance was evaluated based on its ability to remove hair artifacts effectively while preserving the integrity of the underlying melanoma lesions.

#### 3.2.1 Hair removal efficacy

In tests against the synthetic hair dataset, the model achieved a high precision rate in identifying and removing hair artifacts, with minimal instances of mistakenly altering lesion areas. The removal process did not introduce noticeable distortions or artifacts, preserving the essential diagnostic features of the melanoma lesions. The model’s ability to maintain the integrity of lesion details post-hair removal was confirmed through the SSIM and visual inspections, which verified that lesion morphology, coloration, and border characteristics were successfully preserved.

After adjusting the weights for each loss component, the model underwent retraining with specifically allocated weights to optimize the balance between the loss components, thereby enhancing the output. Hair pixel loss and non-hair pixel loss were prioritized with weights of 2.5 and 3.5, respectively, while assigning lower weights to normalizing hair pixel loss (0.3), SSIM loss (1), and total variation loss (0.5). This calibration process included iterative testing, in which various weight configurations were tested, and the model was retrained multiple times to determine the most effective combination. Each configuration’s performance was rigorously evaluated by analyzing outputs and examining the loss history graph.

**Fig. 4:**
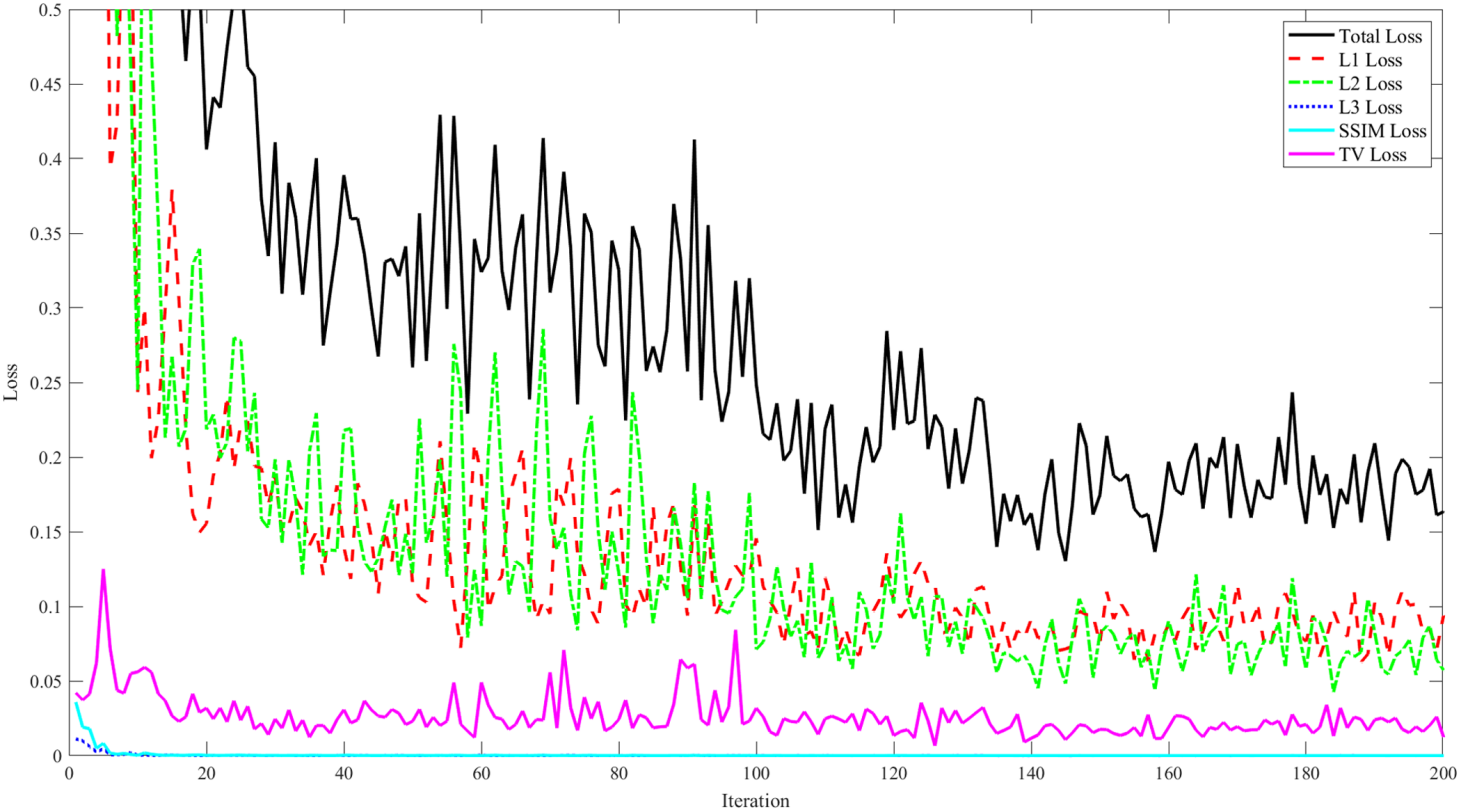
Loss history graph for model training on hair-removed dermoscopic images. Evolution of the different loss components (L1, L2, L3, SSIM, and TV) over 200 training iterations. The black line represents the total loss, reflecting the overall learning process.

This approach, drawing on insights from prior iterations, involved retraining the model using a dataset of 852 dermoscopic images. The results, as depicted in Fig. 4, demonstrate an improvement in the model’s learning process throughout training.

Figure 4 presents a graph representing the loss history, with each of the five loss components plotted over the training iterations. The total loss ensures a comprehensive approach to the hair removal process. The model not only effectively removed hair but also more accurately preserved the integrity of the skin details.

**Fig. 5:**
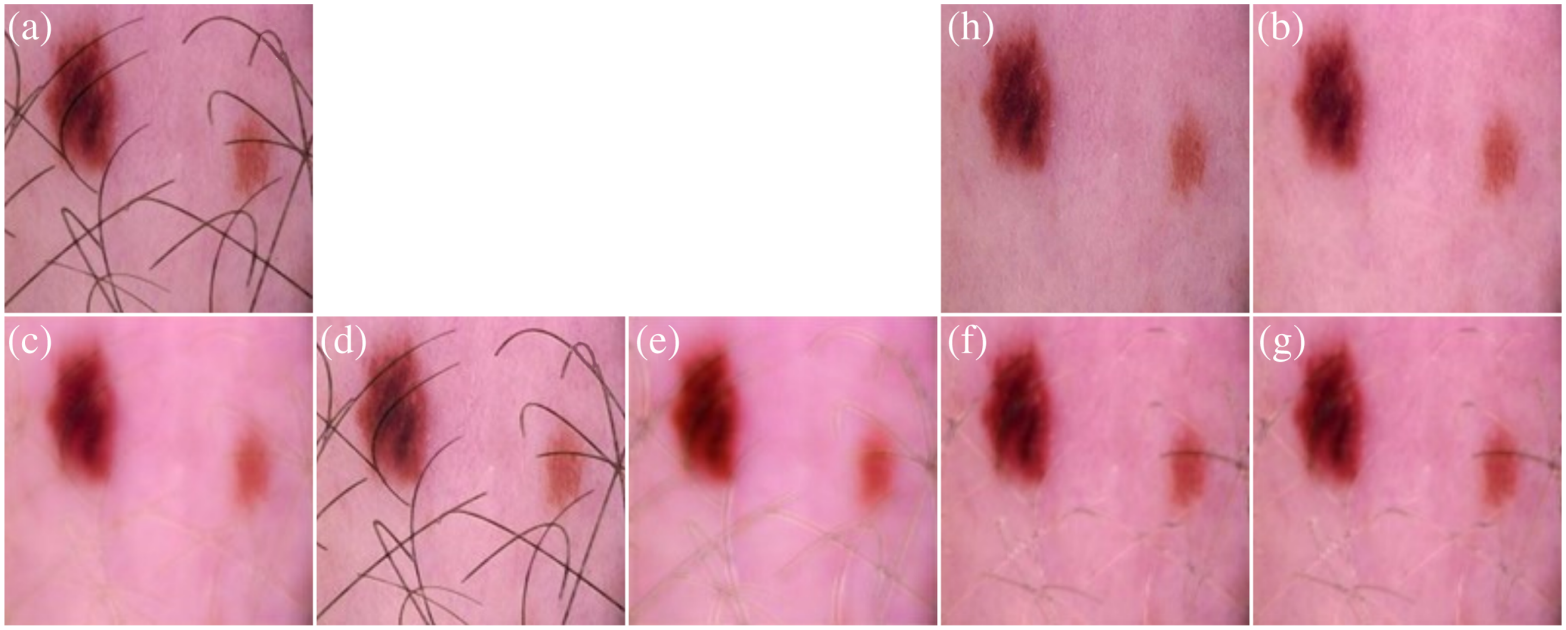
Comparison of hair removal models with the same architecture using different loss functions on synthetic hair. (a) Original dermoscopic image with synthetic hair. (b) Output result of a trained model with combined losses. (c) Output result of a trained model with hair pixel loss. (d) Output result of a trained model with non-hair pixel loss. (e) Output result of a trained model with normalizing hair pixel loss. (f) Output result of a trained model with SSIM loss. (g) Output result of a trained model without total variation loss. (h) Ground-truth image without hair.

Figure 5 presents a comparative analysis of different hair removal methods applied to dermoscopic images. The original image, containing synthetic hair, is shown in subfigure (a). The subsequent subfigures, (b) through (g), display the results from models with the same architecture using different loss functions, each employing a distinct loss function aimed at enhancing the hair removal process.

This comparison highlights the strengths and limitations of each method, illustrating how different loss functions influence the effectiveness of hair removal in dermoscopic images. Image (b) illustrates the output from a model trained using a combination of losses, specifically designed to balance multiple aspects of image quality while focusing on effective hair removal. Image (c) demonstrates the results when the model is optimized primarily with hair pixel loss, concentrating on accurately removing hair pixels. Image (d) shows the output where the focus is on non-hair pixel loss, emphasizing the preservation of non-hair regions to maintain the integrity of the image. Image (e) presents the approach using normalizing hair pixel loss, aiming to normalize the influence of hair pixels across the image to ensure consistency. Image (f) depicts the model’s output using Structural Similarity Index (SSIM) loss, prioritizing maintaining structural similarity between the original and processed images, ensuring that important textural details are preserved. Finally, image (g) provides the result from a model trained without incorporating total variation loss, highlighting the effects of excluding this regularization technique on the overall image quality. This series not only showcases the technical capabilities of each model but also serves as a vital reference for selecting the appropriate loss function based on specific hair removal needs in dermoscopic image analysis.

The precision measures how accurately the model identifies hair without misclassifying lesion details, whereas recall reflects the model’s ability to detect and remove hair artifacts. The F1 score, the harmonic mean of precision and recall, provides a balanced measure of overall model performance. Our model achieved a mean precision of 0.6916, focusing only on pixels near the hair masks to preserve lesion integrity, whereas other models may alter important regions. The mean recall of 0.8678 indicates successful detection of most hair artifacts. The mean F1 score of 0.7699 reflects the model’s overall accuracy in hair removal.

**Fig. 6:**
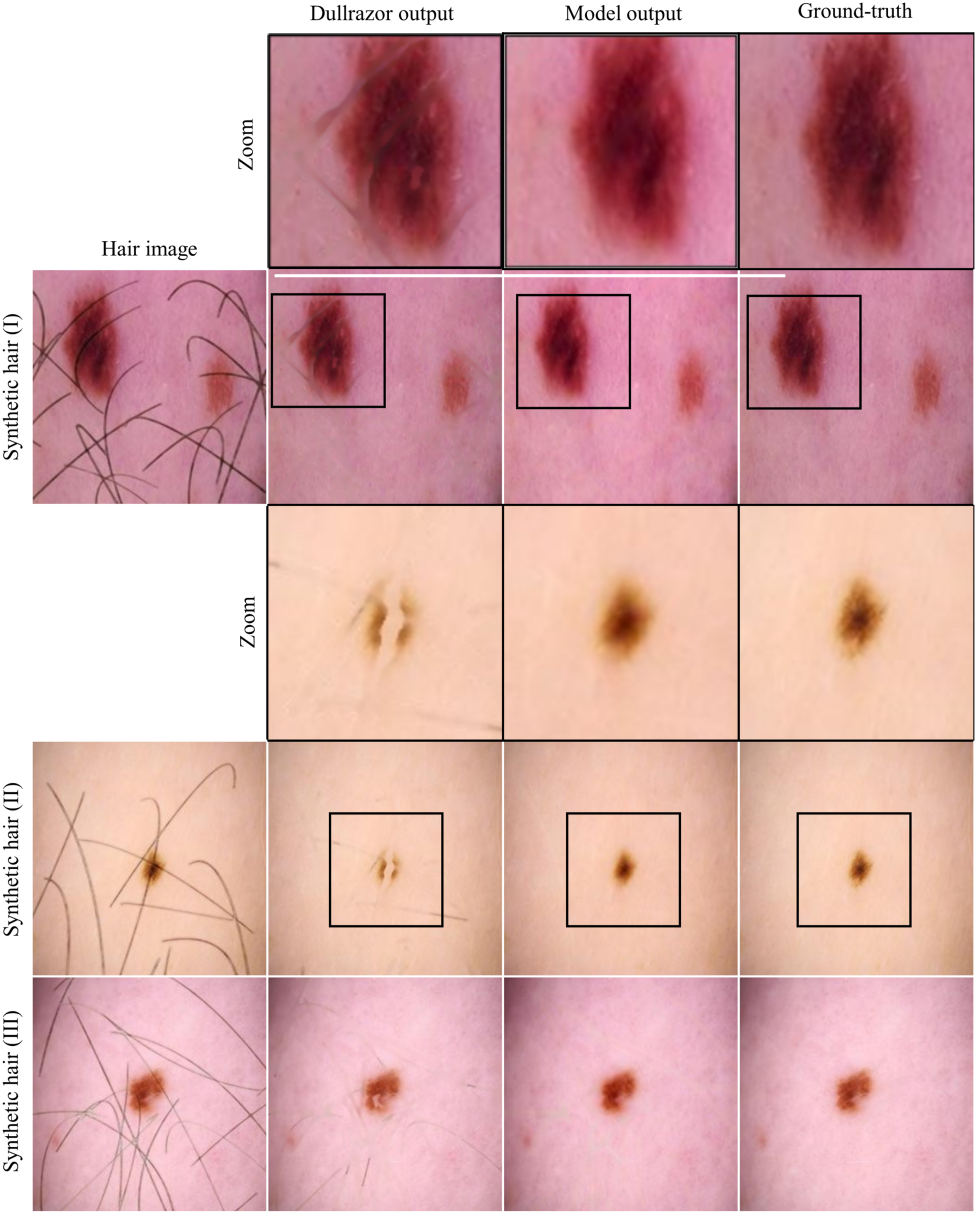
Comparison of hair removal techniques between the presented method and Dullrazor for synthetic hair. In comparison with Dullrazor, the presented method does not leave traces and keeps the lesion integrity intact.

Figure 6 illustrates the visual differences between the traditional Dullrazor method and the proposed method on synthetic hair images, highlighting the improved performance and image quality achieved by the latter.

The method described in this paper demonstrates superior hair removal capabilities for dermoscopic images with synthetic hair when compared with Dullrazor.

In the following, performance metrics are provided for a test set of 100 images when comparing our method with Dullrazor. To achieve this, we prepared an additional dataset of 100 images. Specifically, we selected 10 images from the ISIC archive and generated 10 variants of each by applying different hair masks. Each hair mask contained at least 10 individual hairs, ensuring a diverse representation of hair patterns across the dataset. For each sample, Table 1 demonstrates that our model consistently produces high-quality images. The quality of these images is assessed using the SSIM by comparing them to the original hair-free images.

**Table 1:** Comparison of MSE, MAE, SSIM, and MS-SSIM for Dullrazor and our method with the ground truth (std: standard deviation). SSIM and MS-SSIM: Values closer to 1 indicate better similarity. MSE and MAE: Values closer to 0 indicate better performance.

Image set	MSE		MAE		SSIM		MS-SSIM	
	Dullrazor	Model	Dullrazor	Model	Dullrazor	Model	Dullrazor	Model
**1**	0.0018	0.0174	0.0063	0.1269	0.952	0.947	0.9727	0.955
**2**	0.0084	0.004	0.0076	0.0019	0.9445	0.9834	0.9472	0.9911
**3**	0.0023	0.0015	0.0606	0.034	0.9493	0.9744	0.9612	0.9863
**4**	0.0025	0.0036	0.0609	0.0558	0.9503	0.978	0.9653	0.9851
**5**	0.0039	0.0030	0.0765	0.0533	0.9376	0.9517	0.9658	0.9735
**6**	0.0021	0.0019	0.0601	0.0393	0.9534	0.9675	0.9731	0.9826
**7**	0.0042	0.0036	0.061	0.0538	0.9516	0.9736	0.9703	0.9844
**7**	0.003	0.0015	0.0805	0.0355	0.9419	0.9687	0.9627	0.9834
**8**	0.0059	0.0054	0.0731	0.0683	0.9442	0.9584	0.9724	0.9809
**10**	0.0029	0.0008	0.0657	0.0253	0.9477	0.9736	0.9704	0.9874
**Mean**	0.0037	0.00399	0.05523	0.04941	0.94725	0.96763	0.96681	0.98097

For the SSIM, our method outperforms Dullrazor in nine out of 10 cases. In the one instance where Dullrazor achieves a higher SSIM, the difference in SSIM values is minimal. The average SSIM for the presented method is 0.9676 (std: 0.0038). The average SSIM for Dullrazor is 0.9472 (std: 0.0155). The developed method also produces more consistent results across different hair masks, as reflected by the lower standard deviation of the SSIM compared with Dullrazor. The smaller standard deviation of the SSIM indicates that our model performs more uniformly across different cases, whereas Dullrazor exhibits higher variability. These results demonstrate that the presented method outperforms Dullrazor in terms of structural similarity on synthetic hair. Although Dullrazor is effective in removing hair, it tends to blur lesion details more than our CNN-based approach, resulting in lower SSIM values.

Our model demonstrates superior performance across multiple other metrics: it achieves lower MSE in seven out of 10 image sets, lower MAE in nine out of 10 image sets, and higher MS-SSIM in nine out of 10 image sets.

**Fig. 7:**
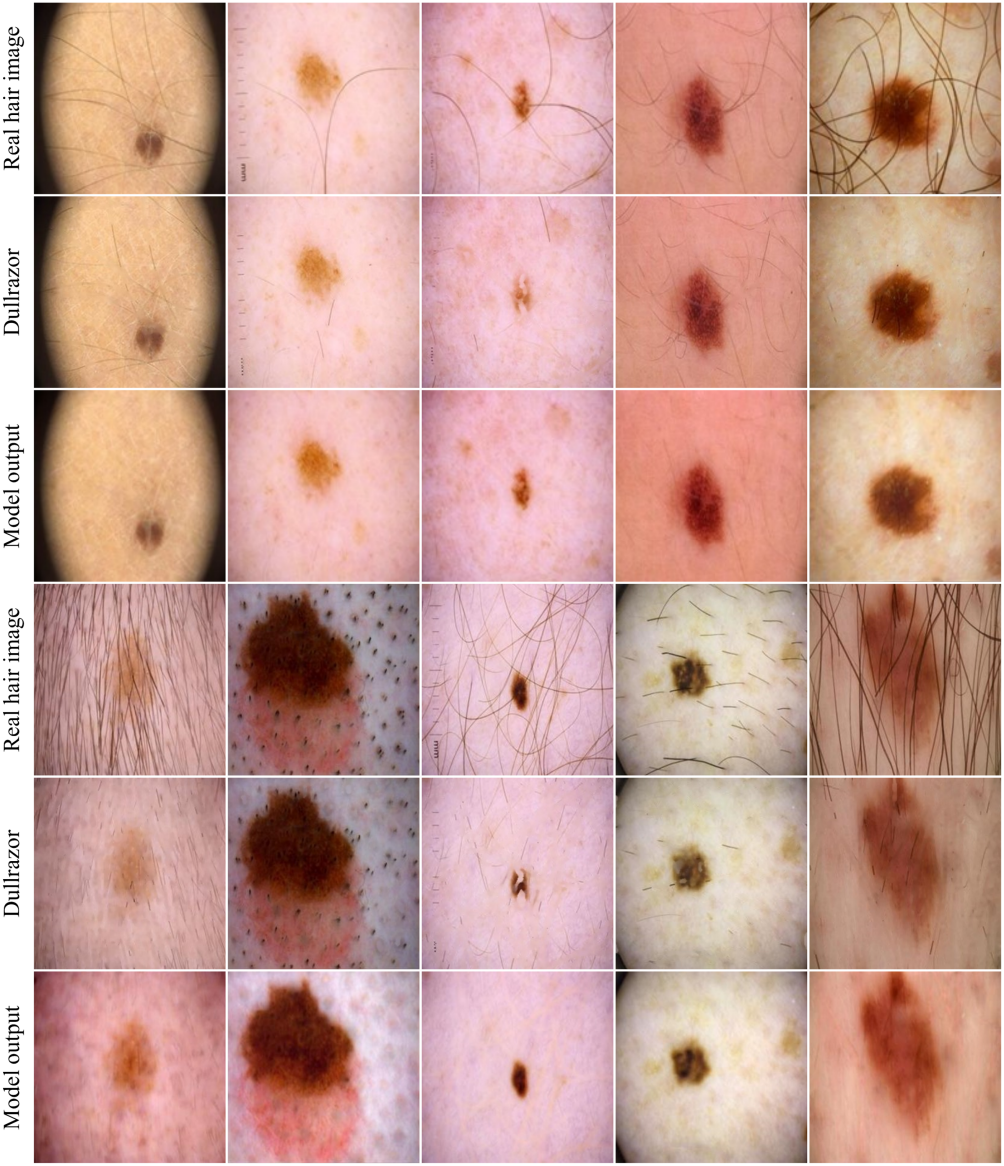
Comparison of the model performance on ISIC data test samples with real hair against the state-of-the-art digital hair removal method Dullrazor.

In Fig. 7, the effectiveness of our hair removal model on real hair is illustrated through a series of images from the ISICData Archive.

The first and fourth rows present the original test sample images with real hair, which display various hair patterns before applying the hair removal algorithm. The second and fourth rows show the hair removal results with Dullrazor. Figure 7 shows that Dullrazor does not reliably detect and remove hair. In some cases, Dullrazor also removes parts of the lesion. The third and sixth rows showcase the results post-processing by our model, highlighting the areas from which hair has been successfully removed without removing any lesion parts. This clear before-and-after comparison underscores the model’s precision and effectiveness in hair removal, essential for aesthetic or clinical applications. The samples were selected to demonstrate the model’s effectiveness in removing hair from dermoscopic images across a wide range of skin tones. This highlights the importance of the diversity of the dataset and confirms that the model is capable of successfully removing hair from different skin types.

The performance metrics for hair removal model evaluation, as shown in Table 2, demonstrate that the model with tuned weights significantly outperforms the baseline across all evaluated parameters, particularly in SSIM and MSSSIM, indicating enhanced structural and textural accuracy. This systematic evaluation allows for the assessment of each weight configuration’s effectiveness based on the calculated metrics, pointing to the potential for further fine-tuning of the loss weights. By continuously adjusting the loss weights based on quantitative feedback, the model’s potential to preserve crucial diagnostic information in processed dermoscopic images can be further enhanced, optimizing its utility in clinical settings.

**Table 2:** Performance metrics for hair removal model evaluation (ideal value 1.0).

Metrics	Model with uniform weights ($\lambda_{i}=1$)	Model with initial weights ($\lambda_{1,2}=0.5$, $\lambda_{3,4,5}=0.2$)	Model with tuned weights ($\lambda_{1}=2.5$, $\lambda_{2}=3.5$, $\lambda_{3}=0.3$, $\lambda_{4}=1$, $\lambda_{5}=0.5$)
**MSE**	0.978941	0.993771	0.994203
**MAE**	0.879822	0.923138	0.931803
**SSIM**	0.840377	0.895211	0.947309
**MSSSIM**	0.873124	0.879974	0.982331

**Fig. 8:**
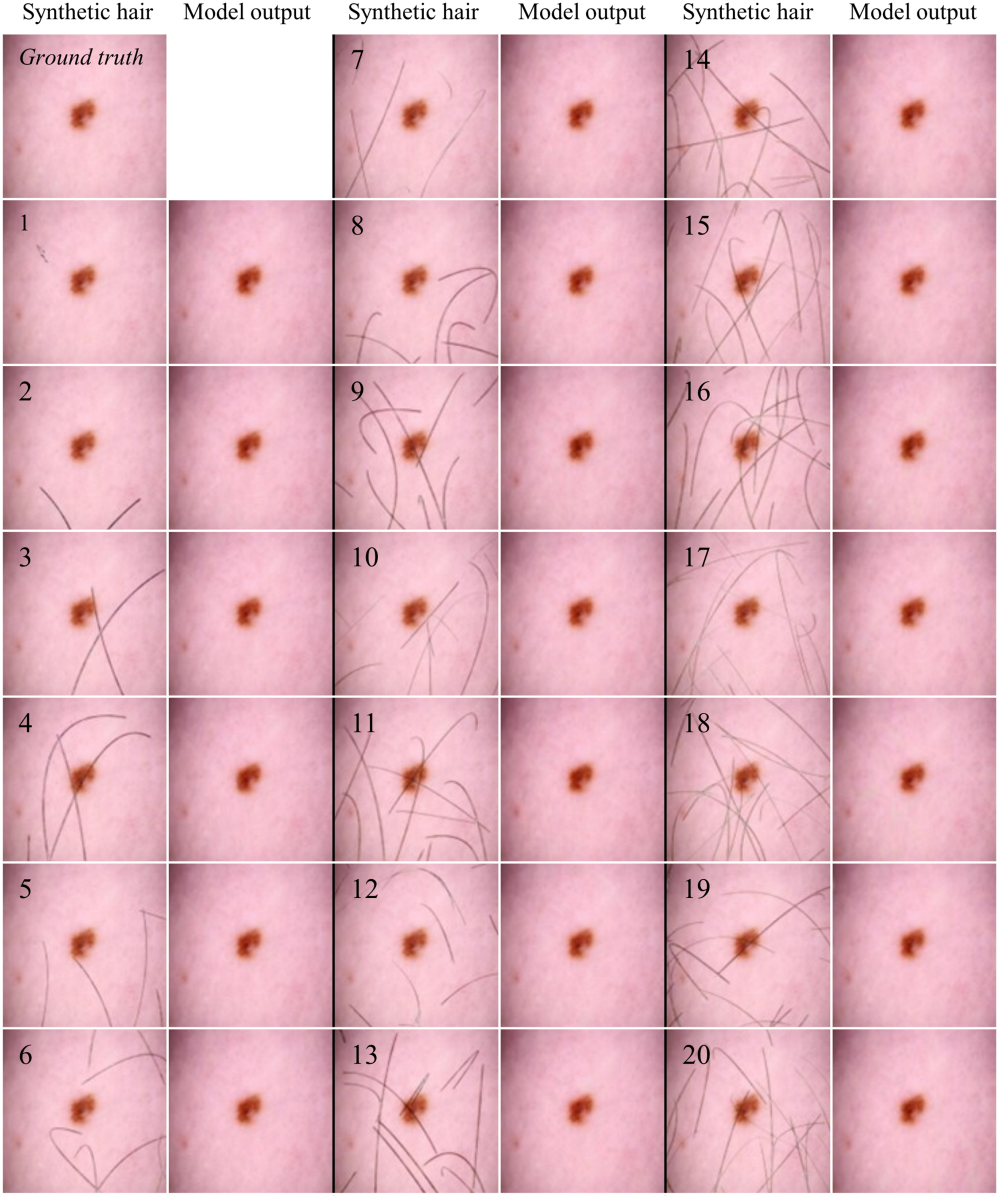
Performance of hair removal model on images with increasing synthetic hair coverage.

In our experiments, we applied 20 masks to 10 sample images to assess the performance of the hair removal model. Each mask incrementally added more hair, with the first mask having one hair and the last mask having 20 hairs as displayed in Fig. 8.

**Fig. 9:**
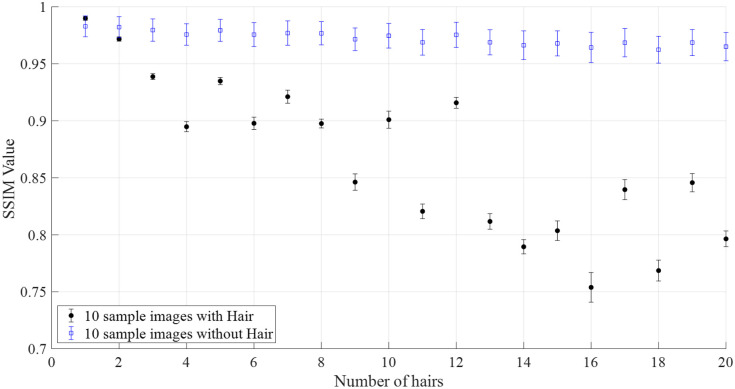
Comparison of mean SSIM values with increasing number of synthetic hairs. The error bars show the standard deviation of 10 samples.

Initially, synthetic images with less hair coverage had a higher SSIM compared with the ground truth, which significantly improved after hair removal. Conversely, images with more extensive hair coverage started with a lower SSIM, but still showed a substantial increase after hair removal. The results demonstrate that the hair removal model effectively enhances image similarity, though its performance varies with the amount of hair present. Figure 9 shows the SSIM values for each of the 20 masks applied to a sample image, showcasing the output of our trained model and its effectiveness from minimal to extensive hair coverage.

## 4 Conclusion

This study represents an advancement in the field of dermatological imaging, particularly in the early detection and diagnosis of melanoma through dermoscopy. The research set out to address the considerable challenge posed by hair artifacts in dermoscopic images, which can obscure critical details of melanoma lesions and potentially lead to misdiagnoses. By creating a comprehensive synthetic hair dermoscopic image dataset and developing a specialized deep learning model for hair removal, this study has introduced innovative solutions that enhance the clarity and diagnostic utility of dermoscopic images.

The synthetic hair dataset provides the simulation of a wide array of hair types, colors, and densities and can be a resource for the development and evaluation of hair removal algorithms. This dataset not only provides a novel tool for the dermatological community but also sets a new benchmarking standard for research in dermoscopic image analysis. It enables robust training and testing of algorithms, ensuring their effectiveness across the diverse scenarios encountered in clinical practice.

The deep learning model developed for hair removal, leveraging advanced CNN architecture, demonstrated great performance in removing hair artifacts while preserving essential lesion details. This balance is important for maintaining the diagnostic integrity of dermoscopic images. The model’s efficacy was validated through extensive testing against both the synthetic hair dataset and dermoscopic images with real hair, showcasing its ability to significantly enhance image clarity. The comparative analysis with existing hair removal methods further highlighted the superiority of the developed model in terms of hair removal completeness and lesion detail preservation.

Despite the results achieved, this research has also indicated areas for further investigation and improvement. The generalization capabilities of the deep learning model across a broader spectrum of real-world images, the optimization of processing times, and the reduction of computational requirements are among the challenges that future work will aim to address. In addition, the integration of the model into clinical workflows, ensuring it complements and enhances dermatological practice without introducing significant burdens, remains a critical objective. The development of non-contact dermoscopy technologies offers potential solutions to these challenges. For instance, the use of an ultra-bright light source and a liquid lens-based autofocus function in non-contact devices enhances feature resolution and color reproducibility, crucial for the differential diagnosis of skin conditions beyond cancer.^26^ Furthermore, the implementation of focus stacking techniques in non-contact dermoscopy ensures all-in-focus imaging, which is essential for capturing accurate topographical details of skin lesions.^27^ These advancements demonstrate significant improvements in the clarity and diagnostic utility of dermoscopic images, irrespective of patient skin type or the presence of superficial obstructions such as hair.^28^

Although there are other synthetic hair datasets, they are limited in size, which is not sufficient for machine learning. Comprising over 1000 image pairs, our dataset is adequate for machine learning approaches. Our model can simulate a wide variety of hair types, densities, and skin colors with real melanoma lesions, offering a more realistic and comprehensive resource for training hair removal models. Furthermore, with the presented approach it is possible to generate training datasets for specific needs, for example, only images with low hair density or only images with thick hairs could be generated with the developed hair generator.

We plan to collaborate with dermatologists to conduct clinical case studies, in which our hair removal model will be applied to real dermoscopic images in a clinical setting. These case studies will assess how the hair removal process enhances diagnostic clarity and whether it improves the dermatologist’s ability to detect melanoma, particularly in challenging cases where hair coverage obscures critical features. In addition, we will include feedback from dermatologists using the model in clinical practice to gather qualitative data on its effectiveness in improving diagnostic confidence, further validating its impact in clinical settings. The dermatologists’ feedback could be used to further refine the model, ensuring that it meets the practical needs of clinicians. Adjustments could include tuning the model to handle specific clinical conditions.

In conclusion, this paper contributes to the advancement of early melanoma detection and diagnosis in dermatology. Addressing the challenge of hair artifacts in dermoscopic images, not only enhances the clarity and utility of these images but also paves the way for more accurate and reliable melanoma diagnoses. It is important to discuss its limitations with concrete examples. The hair removal model effectively enhances image similarity to the ground truth. Images with less hair significantly improve, whereas those with more hair also see a notable increase. Although the model consistently improves similarity, its performance varies with the amount of hair coverage. The synthetic hair image benchmark dataset and the deep learning solution for hair removal developed in this research hold promise for improving patient outcomes through the early detection and treatment of melanoma.

## Data Availability

Data is available upon reasonable request.
